# Reasons for Increasing Daily Methadone Maintenance Dosage among Deceptive Patients: A Qualitative Study

**DOI:** 10.25122/jml-2020-0038

**Published:** 2020

**Authors:** Mohsen Khosravi, Rashya Kasaeiyan

**Affiliations:** 1. Department of Psychiatry and Clinical Psychology, Zahedan University of Medical Sciences, Zahedan, Iran; 2. Department of Clinical Psychology, Shiraz University of Medical Sciences, Shiraz, Iran

**Keywords:** Maintenance, methadone, deception, patients, treatment

## Abstract

Methadone maintenance treatment might be commonly associated with lying for several causes or manipulating psychiatrists and therapists by a number of patients. Deceptive patients tend to manipulate their psychiatrists for multiple causes. This study aims to improve clinicians’ therapeutic decision-making by identifying the reasons for increasing daily methadone maintenance dosage among deceptive patients. One hundred ninety-six patients undergoing the Methadone Maintenance Treatment (MMT) with no statistically significant difference between the overall Addiction Severity Index (ASI) scores at different doses of methadone (< 60 mg/d and ≥ 60 mg/d) and Ahvaz Reality Distortion Inventory (ARDI) scores > 30, were examined in the current qualitative study with multiple semi-structural interviews about the reasons for increasing daily methadone maintenance dosage. The investigation results revealed that the most common reasons for increasing daily methadone maintenance dosage among deceptive patients were opium craving, patient willingness to feel euphoria, fear of the withdrawal signs, earn money through the sale of surplus methadone, improve the symptoms of physical and psychiatric comorbidity, forgetting painful memories, curiosity, the influence of others, sexual issues, feeling of well-being, and appearance changes. Given these reasons, any increase in daily methadone maintenance dosage is not necessarily accompanied by improvement in the clinical condition of patients. However, clinicians can make the most appropriate therapeutic decision by putting the psychological assessments and clinical interviews into play.

## Introduction

Methadone maintenance treatment might be commonly associated with lying for several causes or manipulating psychiatrists and therapists by a number of patients. Manipulation means deliberately developing or exaggerating the symptoms. Although psychiatrists are trained to collect the information vigilantly, they are likely to be manipulated. Recent studies have indicated that even experienced psychiatrists may fail to detect malingering in some cases. Patients tend to manipulate their psychiatrists for multiple causes. Sometimes they are motivated by secondary gains such as work exemptions, disability confirmation, or obtaining their interested medications. Others may seek primary gains like the psychological advantages of accepting the patient role [1]. Unfortunately, no reliable tools have been developed to detect manipulation so far. However, during the interview process, certain symptoms of the patient, including inconsistent, ambiguous responses, incompatible symptoms and performance, and unreasonable claims on a given mental condition, will cast doubts on their behavioral authenticity [1, 2]. It should be noted that the high comorbidity of personality disorders in people with substance use disorders complicates the clinical condition (in particular, the antisocial and borderline personality disorders) [3].

Understanding the reasons for increasing daily methadone maintenance dosage among deceptive patients is important for two causes. Firstly, during the therapeutic decision-making process, psychiatrists confront two issues of trust and distrust in their deceptive patients’ complaints. These issues may lead to devastating effects by increased and steady constant or decreased daily doses of methadone maintenance. In this regard, knowing the reasons for increasing daily methadone maintenance dosage among deceptive patients can help trust some patients’ complaints and find the most appropriate treatment for them. The increased methadone dosage (particularly higher than 60 mg per day) has led to more complications, including QT interval prolongation, heart conduction disorders, osteoporosis, infertility, overdose, and chronic gastrointestinal complications (especially constipation) [4, 5]. However, unawareness of the patients’ needs may lead to the destruction of the faith of patients in their physicians and a reduction in medication adherence [4, 6]. Secondly, unconsciousness is another important topic during the therapeutic decision-making process. From the psychodynamic and neurobiological perspectives, many mental activities are performed unconsciously. In other words, both the psychiatrist and patient act both consciously and unconsciously, bringing their personality characteristics, personal histories, and idiosyncrasies to the interview process. Conscious and unconscious processes are exhibited in the patient-physician relationship through transference, countertransference, and different defense mechanisms. Such deviations result in unhelpful behaviors and emotions in the patient and physician, eliminating the therapeutic opportunities. Therefore, knowing the patients’ reasons for increasing daily methadone maintenance dosage by adopting a non-judgmental attitude towards them and correctly identifying their needs may greatly contribute to the improved patient-physician relationship as well as enhanced medication adherence [6, 7].

By investigating the reasons for increasing daily methadone maintenance dosage among deceptive patients, this qualitative study attempts to help improve the patient-physician relationship and obtain a more effective understanding of this patient group’s physical and psychological needs as mitigating the processes of transference and countertransference.

## Material and Methods

### Sampling and study setting

In this qualitative study, the convenience sampling technique was used to collect the samples within a one-year period. During that period, after giving the necessary explanations about the purpose and methods, as well as following the principles of the Declaration of Helsinki, all the patients aged 18 to 50 who referred to rehabilitation centers of Zahedan and treated with less than 60 mg of methadone per day during the last month, were evaluated using the Addiction Severity Index (ASI). The exclusion criteria of the current study were: 1) Significant statistical difference between ASI scores of < 60 mg daily methadone consumption and ≥ 60 mg; 2) Illiteracy; 3) Severe physical and psychological diseases (like psychosis and bipolar disorders), which need immediate interventions; 4) Positive urine morphine test, two times in a row within two weeks or more than one week of absence; 5) Being homeless; 6) Intellectual disability; 7) Prisoners or immediate imprisonment; 8) Use of an illegal drug and medication.

### Procedure

**Figure 1:**
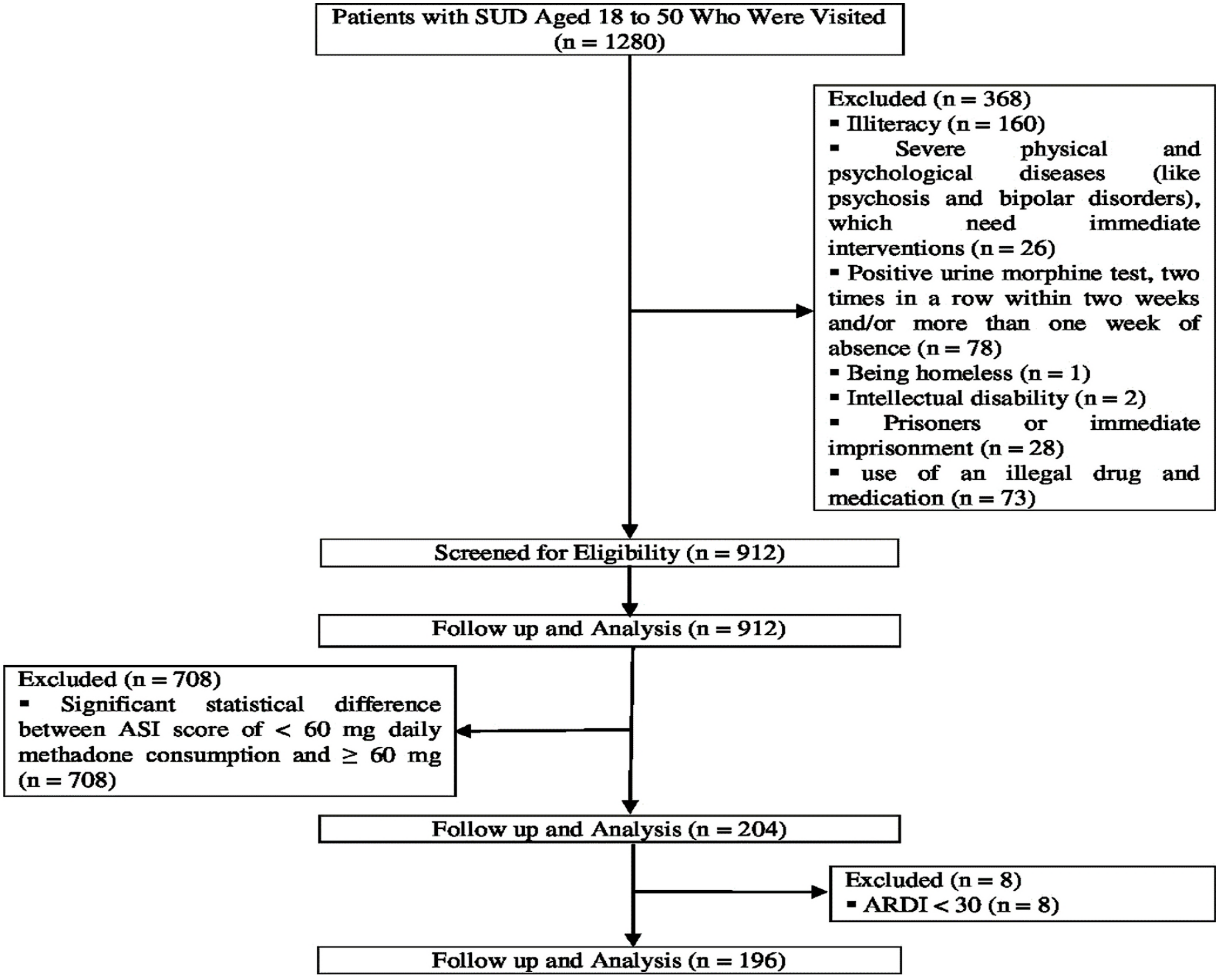
The CONSORT flow diagram of the study.

After evaluating 912 patients undergoing methadone maintenance treatments with ASI, 204 persons who increased methadone maintenance doses from < 60 mg to ≥ 60 mg, but with no significant difference in mean ASI scores with different amounts of methadone (< 60mg or ≥ 60mg), were selected. The Ahvaz Reality Distortion Inventory (ARDI) was used to confirm the deception of these patients. Only patients with ARDI scores > 30 were entered into the study (Figure 1). Then, the patients were evaluated through multiple semi-structured interviews on the reasons for increasing daily methadone maintenance dosage based on the framework presented in Table 1. A large number of clinical interviews was planned to examine changes over time. However, the interviews were stopped at 196 when the investigator concluded that the saturation had been reached, and no new information was being generated (Tables 2, 3).

**Table 1:** Framework of semi-structured interviews.

**1.** Multiple semi-structured interviews (at least 3 times) with participants ranging from 20 to 40 minutes. In order to evaluate controversies in patients’ statements, they were asked at the beginning of sessions to retell what they have told previously. For example, every participant was told: “can you tell me again what you have told me in the previous sessions ?”
**2.** Gathering data and making inquiries about the clinical condition of patients, their physical condition, function level, and symptoms of withdrawal from collateral sources.
**3.** Confronting subjects that participants have given incorrect information about. In this regard, questions were asked in a way that avoids accusing the patient. For example: “somethings make me confused; you said that you have never had problems in this area, while this is not compatible with what you have told me at the beginning of the last session. Can you help me understand this?”

**Table 2:** Frequency of demographic variables based on descriptive statistics.

Demographic variables	Frequency	Percent
**Male**	167	85.2
**Female**	29	14.8
**Non-degree**	84	42.9
**High school diploma**	67	34.2
**Academic degree**	45	23.0
**Single**	95	48.5
**Married**	75	38.3
**Divorced**	26	13.3
**Total**	196	100
Mean age	Mean ± SD	
	34.7 ± 8.1 years old	

**Table 3:** Comparison of the ASI scores in different areas within times A and B using the independent t-test and total mean ARDI score.

ASI areas	Methadone dosage	Mean ± SD	T	Df	p
Medical condition	< 60 (A)	0.70 ± 1.02	-2.29	1	0.140
	**≥ 60 (B)**	1.03 ± 0.99			
Substance use condition	< 60 (A)	2.03 ± 0.85	-2.0	1	0.254
	**≥ 60 (B)**	2.23 ± 0.75			
Legal condition	< 60 (A)	0.63 ± 0.99	0.24	1	0.852
	**≥ 60 (B)**	0.60 ± 0.70			
Employment	< 60 (A)	1.56 ± 0.77	2.23	1	0.136
	**≥ 60 (B)**	1.35 ± 0.57			
Family condition	< 60 (A)	2.50 ± 0.77	1.65	1	0.294
	**≥ 60 (B)**	2.32 ± 0.75			
Psychological condition	< 60 (A)	1.73 ± 1.14	-0.06	1	0.983
	**≥ 60 (B)**	1.74 ± 1.04			
Total mean ASI score	< 60 (A)	0.58 ± 1.53	-0.28	1	0.867
	**≥ 60 (B)**	0.45 ± 1.55			
ARDI score	Minimum	Maximum	Mean ± SD		
	**31.00**	40.00	35.12 ± 2.85		

The study started with several general questions on the subject. While designing the questions, previous literature with the comments of experts and faculty members were considered. Some of the questions had previously been designed based on the objective of the study, while other deep and exploratory questions (e.g., questions asking for explanations or on why/how) were devised based on the answers of the participants. The interviews with the patients and their companions were performed in the hospital, rehabilitation centers, or locations selected by them, including the hospital courtyard and the waiting rooms. The interviews were audio-recorded and transcribed verbatim. In order to analyze the obtained data, an analysis framework based on five steps of familiarizing, identifying a thematic framework, indexing, charting, and mapping, as well as interpreting was used. The content of each interview was written down within 24 hours. In this analysis, after familiarization with the range and diversity of the contents, the researchers identified the key concepts and topics based on which a conceptual framework was later designed. Next, all the texts corresponding to the individual interviews were reviewed and revised 2-4 times based on the obtained conceptual framework, followed by organizing the texts according to the most appropriate conceptual source. The concepts, conflicts, theories, experiences, and previous studies were also compared to each other, and the desired patterns and relations were extracted from the findings.

Along with the data collection process, the coding process was also performed. The main concepts were defined in the form of initial codes. In the next stage, the codes with similar concepts were put together to create secondary classes using the MAXQDA software, version 18.

## Measurements

### 

#### Addiction Severity Index (ASI)

The truncated version of the ASI scale consists of 106 questions covering six areas, namely the medical, substance use, employment, family, legal, and psychological conditions of the respondent. It measures the professional, family, legal, and psychological conditions of the respondent in the past 30 days, as well as the dosage, term, and severity of substance use. The questions are ranked based on the 5-point Likert scale (0-4). In Iran, Cronbach’s alpha coefficient of this questionnaire was between 0.65 and 0.89 [8].

#### Ahvaz Reality Distortion Inventory (ARDI)

The ARDI questionnaire consists of 10 questions. Participants should choose one of the four options for each question. The total score of ten materials shows the distortion rate of reality for each subject. Earning more than 30 points in ARDI indicates a higher distortion of reality. Answers to questions take place in a framework of a 4-point Likert scale (1-totally disagree to 4-strongly agree). Its internal consistency through Cronbach’s alpha coefficient was 0.75 [9].

#### Reasons for increasing daily methadone maintenance dosage based on participants’ viewpoints

Based on the view of the interviewees, two categories of personal factors (mental beliefs, economic reasons, as well as physical and mental reasons) and social factors (family, friends, and colleagues), eleven themes of opium craving, patient willingness to feel euphoria, fear of the withdrawal signs, earning money through selling the surplus methadone, improving the symptoms of physical and psychiatric comorbidity, forgetting painful memories, curiosity, the influence of others, sexual issues, feeling of well-being, as well as appearance changes, as well as 21 subthemes were obtained concerning the increase in the daily dose of methadone maintenance among the deceptive patients.

### Theme 1: Opium craving

#### Affordability and availability of the drugs

Example: “Whenever I went, someone was abusing drugs. There were even some people in the market who whispered prices for various drugs much cheaper than methadone. It seemed that my eyes were only seeing addicts, and my ears were only hearing the name of different drugs. I was always afraid of slipping up again” (Male, 28 years old).

#### Weak volition

Example: “I know that I am a weak person. So far, I have quit drugs many times, but I have always come back to them. I am tired of all of these. I did not want to fell in the trap of drugs once again” (Male, 22 years old).

### Theme 2: Patient’s willingness to feel euphoria

#### Feeling boredom and fatigue after quitting drugs

Example: “After quitting drugs, I was so bored and tired that I was not in the mood for doing anything. I tried to feel better by increasing the dose of my methadone” (Male, 24 years old).

#### Methadone as an alternative for experiencing euphoria caused by taking drugs

Example: “The euphoric pleasure after taking a drug was the only reason persuading me to abuse drugs again more than ever before. After quitting drugs, I loved to experience this euphoric feeling, this time with methadone” (Female, 20 years old).

#### Having a novelty-seeking personality trait

Example: “Taking methadone has a pleasure that is temporary, but for reaching that brief pleasure, a man likes to use it more and more” (Male, 31 years old).

### Theme 3: Fear of withdrawal signs

#### Belief in the inability to tolerate withdrawal signs causing relapse

Example: “I do not like to go back to those days. I mean the days I was in withdrawal. It was so painful that I cannot even experience it one more time. In reality, from the day I quit the drugs and started methadone, I was always afraid of feeling so much pain so that I have to return to the drugs. I didn’t have any other option but to trick my doctor into increasing my dose of methadone” (Male, 28 years old).

### Theme 4: Curiosity

#### Seeking diversity trait

Example: “The reality is that I wanted to see how much methadone in a day can give more pleasure to an individual” (Male, 25 years old).

#### Lack of entertainment

Example: “When I was an addict, I would spend most of my time abusing drugs; now that I have quit drugs, I do not have any other entertainment but the methadone” (Female, 20 years old).

### Theme 5: Appearance changes

#### Weight loss and facing changes

Example: “Abusing drugs messed me up. I have heard from others that taking methadone can increase your weight. I thought if I increased my dose, I would gain more weight quickly because I was totally embarrassed with my appearance” (Male, 30 years old).

### Theme 6: Earning money through the sales of surplus methadone

#### Unemployment

Example: “My main problem and difficulty is unemployment. If I had a job, I would not be forced to lie to make a living” (Male, 28 years old).

#### Cheaper governmental methadone

Example: “To be honest, the difference between the free-market and the publicly-funded methadone was very high; thus, I realized I could buy the methadone from the government and sell it in the black market. In this way, I could make a living and also pay for my methadone” (Male, 32 years old).

### Theme 7: Improving the symptoms of a physical and psychiatric comorbidity

#### Easy physical and mental benefits including improvement of depression, anxiety, irritability, headache, insomnia, and restlessness

Example: “The pressure to study was very high. I was highly stressed. When I increased the dose of methadone, I felt my anxiety decreases and I also felt learning is much easier for me” (Male, 19 years old). Another patient said: “Nothing can improve my headache like methadone; it is like water poured on the fire” (Female, 26 years old).

### Theme 8: Forgetting painful memories

#### Childhood traumas, including physical, sexual, and emotional abuse

Example: “I have so many bad memories in my life that even recounting them pains me. The beatings I received from my father... being humiliated in front of others. Nothing is important anymore. I only want to take a drug and forget everything” (Male, 20 years old).

### Theme 9: Sexual issues

#### Increasing arousal and duration of sexual acts

Example: “When I stop taking methadone, I cannot have sexual relations; I do not have the physical endurance to do it. I have to take more methadone to be able to increase the duration of the sexual relation” (Male, 32 years old).

### Theme 10: Influence of others

#### Lack of life skills, including the skill of saying no

Example: “When my friends were betting on how they can trick their doctor into increasing their dose of methadone, I was too embarrassed to disagree with them” (Male, 21 years old).

#### Desire to be different

Example: “I was hiding this from my doctor and my family, but I liked to show my friends that I am responsible for my own life and no one can control it” (Male, 18 years old).

#### Friends influence and peer pressure

Example: “My friends were always encouraging me to consume more methadone. They said consuming a low dose of methadone is not different from not consuming it at all” (Female, 19 years old).

#### Fear of losing job

Example: “I am a manual worker. In order to increase my physical capability for doing more work, I decided to increase the dose of my methadone. When I consumed more methadone, I did not get tired” (Male, 29 years old).

### Theme 11: Feeling of well-being

#### Cultural conditions

Example: “When I see that all my friends and family members are taking one type of illicit drug, I am free to take as much methadone as I like; nothing is more important for me than having a good mood and enjoying my time” (Female, 25 years old).

#### Social status

Example: “In our house, everybody is always fighting. You cannot have any peace of mind. I have to resort to anything to escape this environment” (Male, 25 years old).

#### Having a better life after quitting

Example: “When I was an addict, I was under immense pressure from others; now, I think I can take the methadone as much as I like because there is no pressure from others or the law. It seems that you have great support to consume as much methadone as you like and have fun” (Male, 35 years old).

## Discussion

**Figure 2:**
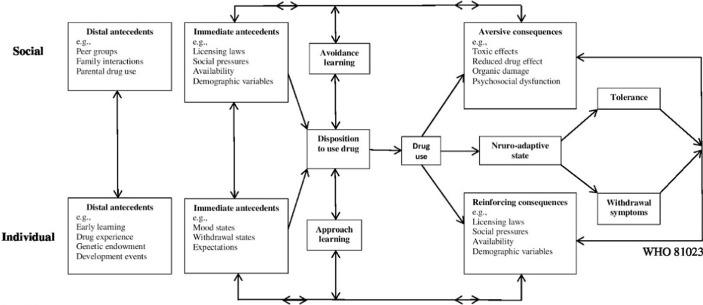
World Health Organization schematic model of drug use and dependence.

The present qualitative study demonstrated increased daily methadone intake of deceptive patients due to two categories of factors: personal (mental beliefs, economic reasons, as well as physical and mental reasons) and social (family, friends, and colleagues), comprising opium craving, patient's desire for euphoria, fearing of withdrawal signs, earning money by selling surplus methadone, improving the symptoms of psychiatric and physical comorbidity, curiosity, forgetting painful memories, sexual issues, the influence of others, well-being feeling, and appearance changes, which is almost in line with the World Health Organization schematic model of drug use and dependence. Based on this model, multiple social factors (such as peer groups, family interactions, parental drug use, availability, demographic variables, and social pressures) and individual factors (such as genetic endowment, drug experience, early learning, expectations, withdrawal states, mood status and developmental events) are involved. Through developing a neuro-adaptive state, according to the model, chronic substance use creates tolerance and withdrawal symptoms, resulting in gradually increased use of substance due to the reduced drug effect as well as the aversive consequences (such as toxic effects, organ damage, and psychosocial dysfunction (Figure 2) [10].

This can be explained by the fact that the process of deciding to use the substances is influenced by the immediate social and psychological conditions as well as the person's previous history. Indeed, substance use initiates a series of consequences with the capability of being rewarding or aversive. The consequences result in increased or decreased probabilities of behavior repetition through the learning process. If the aversive quality of the withdrawal syndrome is above a certain threshold, it will provide a clear recurring motivation for more substance use. Moreover, sensitization of motivational systems enhances the significance of the drug-related stimuli. On the other hand, casual or compulsive substance use is a behavior continuously encouraged by its consequences. Using a substance can enhance its antecedent behaviors by relieving the annoying or unpleasant states such as pain, anxiety, or depression. Regardless of its medication effects, substance use leads to rapid positive reinforcement through achieving the friends' approval or a certain special status in some social situations [11, 12].

Nonetheless, knowing the reasons for deceptive patients to increase methadone dosage can be important in several ways. Firstly, there have been various adverse consequences for prescribing an increased daily dosage of methadone maintenance treatment by the psychiatrist for deceptive patients that include: 1) Drug side effects [13, 14]; 2) Further withdrawal symptoms followed by a decrease in methadone dosage due to a compensatory-balancing mechanism [15]; 3) High risk of methadone overdose due to the long but variable half-life of methadone in the range of 5-55 h [16, 17]. Secondly, unusual behaviors of the deceptive patients bring problems in the patient-physician relationship and pose dangers for the patients themselves. Moreover, psychiatrists and patients should interact necessarily with some degree of reliance. The psychiatrist relies on interviews, which are regularly evidence of the patient's account of what is wrong contributed to the relative history-taking and mental status examination. Afterward, the psychiatrist relies on the patient for cooperation within the suggested treatment, as well as reporting back on any progress. An ideal interview contains the trust between the psychiatrist and the patient in three areas: competence, testimony, and motive. There is a central trusting stance for the relationship between patient and physician. Honest communication may also be flourished in an environment consisting of some degrees of trust. As a consequence of trust, patients express their concerns with no fear of being disparaged or disbelieved. A deeper patient-physician relationship can be developed over time, facilitated due to the presence of trust. Specific types of care necessarily need such a relationship, including disclosure and sensitive or potentially stigmatized management problems. The trust in a patient validates his/her experiences and recognizes his/her competence. This enriched view causes a patient to incorporate his/her expertise into the best interest conception [18]. Thirdly, conscious and unconscious processes are exhibited in the patient-physician relationship through transference, countertransference, and different defense mechanisms. Such deviations lead to unhelpful behaviors and emotions, such as the reduced burden of unnecessary emotional distress. If this process remains unknown, it may deprive patients (especially deceptive ones) of appropriate therapeutic opportunities by developing abnormal behaviors [6, 7]. For example, psychiatrists may distrust treatment failure reports of patients, likely due to doubting either the accuracy or the proficiency of the treatment. The distrust reaction would be the natural human tendency for distrusting accounts disbelievable based on one's wishes. Like shooting the messenger, this kind of distrust is mainly present when the doctor feels responsible for medical science and getting the patient better, while this surely represents the incompetence of patients. In addition to available ill-health burdens, distrust would be an unfair burden leading to hostility as well as inhibiting proper clinical care [18]. Finally, it can be deduced that understanding the multiple reasons for increasing daily methadone maintenance dosage may help the physician better understand the physical and psychological needs of deceptive patients and provide better therapeutic approaches by controlling transference, countertransference, and defense mechanisms and subsequently improving the patient-physician relationship.

The present study has exposed some limitations, regarded as using a convenience sampling technique for sample collection, the relatively small sample size, and the selection of participants only from Zahedan. Based on the findings, increased daily dosage of methadone maintenance among deceptive patients has represented more contextual and local meaning relative to a general and universal meaning. Moreover, the removal of these limitations would improve the precision of the results.

## Conclusion

Psychiatrists are inefficiently trained to elicit the information well and be aware of deceptive patients. The optimal methadone dosage can be determined for the methadone maintenance treatment, possibly through psychological assessments and clinical interviews to analyze patients psychiatrically. The daily dosage of methadone can then be calculated based on the results, which prevent possible mistakes by the clinicians. As a consequence, this might minimize the side effects caused by methadone overuse, facilitating withdrawal in the future. Taking everything into account, providing the non-substance-related and comorbid disorders with mental training and pharmaceutical and non-pharmaceutical adoption has been impressive in reducing daily methadone dosage and improving the life quality and performance of patients undergoing the methadone maintenance treatment. Therefore, using the results of this study during the interview process, clinicians can make appropriate treatment decisions.

## Conflict of Interest

The authors declare that there is no conflict of interest.
